# Resurgence of HIV Infection among Men Who Have Sex with Men in Switzerland: Mathematical Modelling Study

**DOI:** 10.1371/journal.pone.0044819

**Published:** 2012-09-14

**Authors:** Ard van Sighem, Beatriz Vidondo, Tracy R. Glass, Heiner C. Bucher, Pietro Vernazza, Martin Gebhardt, Frank de Wolf, Steven Derendinger, André Jeannin, Daniela Bezemer, Christophe Fraser, Nicola Low, the Swiss HIV Cohort Study, J Barth, M Battegay, E Bernasconi, J Böni, HC Bucher, P Bürgisser, C Burton-Jeangros, A Calmy, M Cavassini, M Egger, L Elzi, J Fehr, M Fischer, M Flepp, P Francioli, H Furrer, CA Fux, M Gorgievski, H Günthard, B Hasse, HH Hirsch, B Hirschel, I Hösli, C Kahlert, L Kaiser, O Keiser, C Kind, T Klimkait, H Kovari, B Ledergerber, G Martinetti, B Martinez de Tejada, N Müller, D Nadal, G Pantaleo, A Rauch, S Regenass, M Rickenbach, C Rudin, P Schmid, D Schultze, F Schöni-Affolter, J Schüpbach, R Speck, P Taffé, A Telenti, A Trkola, P Vernazza, V von Wyl, R Weber, S Yerly

**Affiliations:** Chairman of the Clinical and Laboratory Committee; Head of Data Center; Chairman of the Mother & Child Substudy; 1 Stichting HIV Monitoring, Amsterdam, The Netherlands; 2 Division of Communicable Diseases, Swiss Federal Office of Public Health, Bern, Switzerland; 3 Institute of Social and Preventive Medicine, University of Bern, Bern, Switzerland; 4 Basel Institute for Clinical Epidemiology and Biostatistics, University Hospital Basel, Basel, Switzerland; 5 Swiss Tropical and Public Health Institute, Basel, Switzerland; 6 Division of Infectious Diseases, Cantonal Hospital of St. Gallen, St. Gallen, Switzerland; 7 Department of Infectious Disease Epidemiology, School of Public Health, Imperial College London, London, United Kingdom; 8 Institute of Social and Preventive Medicine, University of Lausanne, Lausanne, Switzerland; 9 Medical Research Council Centre for Outbreak Analysis and Modelling, Department of Infectious Disease Epidemiology, Imperial College London, London, United Kingdom; Yale School of Public Health, United States of America

## Abstract

**Background:**

New HIV infections in men who have sex with men (MSM) have increased in Switzerland since 2000 despite combination antiretroviral therapy (cART). The objectives of this mathematical modelling study were: to describe the dynamics of the HIV epidemic in MSM in Switzerland using national data; to explore the effects of hypothetical prevention scenarios; and to conduct a multivariate sensitivity analysis.

**Methodology/Principal Findings:**

The model describes HIV transmission, progression and the effects of cART using differential equations. The model was fitted to Swiss HIV and AIDS surveillance data and twelve unknown parameters were estimated. Predicted numbers of diagnosed HIV infections and AIDS cases fitted the observed data well. By the end of 2010, an estimated 13.5% (95% CI 12.5, 14.6%) of all HIV-infected MSM were undiagnosed and accounted for 81.8% (95% CI 81.1, 82.4%) of new HIV infections. The transmission rate was at its lowest from 1995–1999, with a nadir of 46 incident HIV infections in 1999, but increased from 2000. The estimated number of new infections continued to increase to more than 250 in 2010, although the reproduction number was still below the epidemic threshold. Prevention scenarios included temporary reductions in risk behaviour, annual test and treat, and reduction in risk behaviour to levels observed earlier in the epidemic. These led to predicted reductions in new infections from 2 to 26% by 2020. Parameters related to disease progression and relative infectiousness at different HIV stages had the greatest influence on estimates of the net transmission rate.

**Conclusions/Significance:**

The model outputs suggest that the increase in HIV transmission amongst MSM in Switzerland is the result of continuing risky sexual behaviour, particularly by those unaware of their infection status. Long term reductions in the incidence of HIV infection in MSM in Switzerland will require increased and sustained uptake of effective interventions.

## Introduction

Annual numbers of new HIV diagnoses in Switzerland according to surveillance data were lower in 2010 than in 2000 for all transmission risk groups except men who have sex with men (MSM) [1]. Furthermore, a nationwide genetic linkage study found that the majority of clusters representing ongoing transmission in Switzerland were linked to MSM [2]. The numbers of new notifications of HIV infection in MSM have been increasing since 2000 in several other high income countries [3]. Contemporaneous outbreaks of sexually transmitted infections (STI) among MSM such as syphilis, rectal gonorrhoea and lymphogranuloma venereum support the concern that these new cases represent increasing HIV transmission rather than increased diagnosis of people infected for a long time [3]–[5]. All other things being equal, the incidence of HIV infection would be expected to have fallen in countries with long term unrestricted access to combination antiretroviral therapy (cART), which suppresses viral replication and reduces the sexual transmission of HIV between heterosexual couples with discordant infection status [6]–[8]. Furthermore, associations have been reported between a reduction in estimated measures of ‘community viral load’ and numbers of new HIV infections at the population level in different risk groups and countries [9], [10]. These data have provided support for proposals to expand the use of cART as a preventive strategy [11].

An increase in the incidence of HIV amongst MSM, despite cART for those who are infected and diagnosed, could result from increasing risk behaviour in HIV-negative or HIV-positive men. Levels of unprotected anal sex have increased in Switzerland [12]–[14], as have the numbers of syphilis and gonorrhoea diagnoses in MSM [15], despite regular ongoing health promotion campaigns. In 2008, an innovative nationwide safer sex campaign called ‘Mission Possible’ was carried out [16]. The goal was to interrupt the transmission of HIV during primary infection by having a three month period of 100% adherence to safer sex for all MSM followed by an HIV test and advice about risk reduction. A process evaluation found that awareness of primary HIV infection increased and 11% of MSM surveyed said that they had had only safer sex during the campaign [16].

More frequent HIV testing or greater test uptake, higher numbers of infected MSM from abroad, or prolonged survival, which increases the pool of infected people could also contribute to the higher observed number of HIV-infected MSM. Mathematical modelling studies are a useful tool for investigating the balance between these different and, sometimes opposing effects [17]. When the benefits of cART on infectivity of and survival with HIV were still uncertain, Blower and colleagues showed how optimistic and pessimistic assumptions resulted in divergent predictions of HIV incidence [18]. More recently, in the face of increasing number of new HIV diagnoses in MSM in the Netherlands, modelling studies by Bezemer and colleagues have found that increasing risk behaviour since the introduction of cART, particularly by those unaware that they were HIV-positive, was the most likely explanation for the observed increase in the number of diagnoses [19], [20].

The objectives of this study were: to describe the dynamics of the HIV epidemic in MSM in Switzerland using data from national surveillance and clinical cohort databases; to explore how the epidemic could develop in the future under various hypothetical scenarios; and to test rigorously the dependence of the model predictions on different underlying parameter assumptions in a multivariate sensitivity analysis.

## Materials and Methods

The basic mathematical model structure was that developed by Bezemer and colleagues [19], [20]. Here we present essential features and describe in detail the adaptations that use Swiss data, or involve new analyses. The model parameters are shown in Table S1. In brief, the model describes HIV transmission, progression and the effects of cART using a set of differential equations written in C (Open Watcom Integrated Development Environment Version 1.7). Maximum likelihood methods were used to fit the model simultaneously to available surveillance data about HIV infections and AIDS cases in Switzerland and thus re-construct the epidemic curve since 1980.

### Model Structure

**Figure 1:**
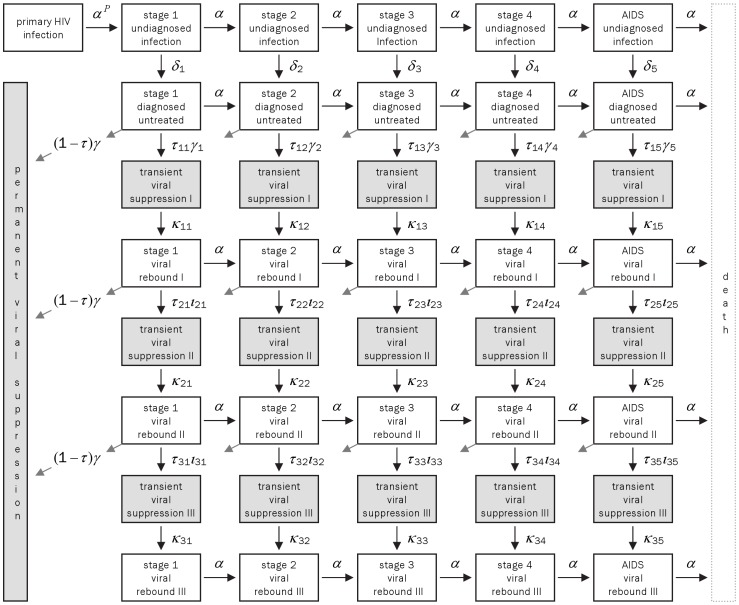
Model structure. Flow diagram of the mathematical model for HIV transmission amongst men who have sex with men (MSM) in Switzerland (adapted from [20]). The model describes progression through different stages of untreated and treated HIV infection. Arrows represent the flow between compartments at rates denoted by Greek letters. Parameter values are given in Table S1.

HIV progression is represented as a unidirectional flow through five compartments (stages) with a mean stay of 1.9 years in each (Figure 1). This structure provides the best fit for an Erlang distribution with degree 5 to the observed survival distribution of MSM seroconverters with untreated HIV infection [20]. The stages do not therefore correspond directly to clinical disease stages or CD4 count categories. Primary HIV infection lasting on average 0.2 years is an extra compartment. HIV infectiousness, relative to that in asymptomatic infection (stages 1 to 4), was estimated to be 25 times higher in the primary stage and 3.2 times higher in stage 5, which was assumed to represent AIDS [19]–[21].

After primary infection, infections in each stage can be undiagnosed or diagnosed. During the cART era, diagnosed infections can be untreated or treated. As a result of treatment, viral suppression is achieved. During viral suppression, it is assumed that no disease progression and, importantly, no onwards transmission takes place. Viral suppression can be either permanent or transient with periods of viral rebound when transmission and disease progression can occur. Viral rebound is assumed to be permanent after three cycles of viral suppression and rebound (equivalent to first-, second- and third-line therapy) and infection then progresses to death from HIV-related causes. We also assumed that there was no loss to follow up.

HIV infectiousness is also modified by knowledge of HIV diagnosis. MSM diagnosed from 1984 to 1996 were assumed to reduce their risk behaviour by 53% after becoming aware of their infection [22]. The model was adapted for this study to incorporate new data suggesting a smaller reduction in risk behaviour following HIV diagnosis of 15% after 1996 [23].

It is assumed that the population susceptible to HIV infection is large, and that removal of the small proportion that becomes infected has a negligible effect on the size of the susceptible population. There is, therefore, no need to introduce susceptibles into the model. The rate of new infections in the model depends on the number of individuals with HIV stratified by disease stage and diagnosis or treatment status [20]. The total rate is calculated as the sum of the number of individuals in each compartment weighted by the relative infectiousness of the compartment multiplied by the net transmission rate.

### Viral Suppression and Rebound in Swiss MSM

The effects of cART on prognosis were modelled using data about rates of viral suppression and rebound. We analysed longitudinal viral load data from MSM diagnosed after 1998 in the Swiss HIV Cohort Study (SHCS) to estimate rates and proportions of viral suppression and rebound at different stages of disease and in different cycles of cART (Table 1). The SHCS data were consistent with the three cycles of viral suppression and rebound in the model. The time from HIV diagnosis or from the start of an episode of viral rebound to achieving viral suppression (HIV RNA <1000 copies/ml) was described using Weibull or log-normal survival models. The time to viral rebound after having achieved suppression was described by ‘relative survival models’ (also known as ‘cure’ models), which allow for a proportion of patients never experiencing viral rebound [24]. For patients with viral rebound, we assumed that progression continued at the same rate as in untreated patients. We also assumed that mono or dual therapy did not slow disease progression. The average rates of viral rebound and suppression incorporate the effects of drug resistance and changes over time in criteria for starting cART. To be able to estimate different rates that could be applied to different stages in the model, we assumed that stages 1–3 corresponded to individuals with CD4>250 cells/mm^3^ at diagnosis, stage 4 corresponded to CD4<250 cells/mm^3^ at diagnosis and stage 5 included all patients with AIDS at diagnosis, irrespective of CD4 count. Model outputs remained almost identical when using 500 copies/ml as threshold for viral suppression although estimates for treatment-related parameters were different (not shown).

**Table 1:** Estimated rates (per year) of viral suppression and viral rebound and fraction experiencing rebound in SHCS participants (n = 1729).

Description of parameter	Value	Range used in sensitivity analysis
Rate of starting therapy and achieving viral suppression in stages 1, 2, and 3, all cycles	0.7	0.6–0.8
Rate of starting treatment and achieving viral suppression in stage 4, all cycles	2.8	2.5–3.2
Rate of starting treatment and achieving viral suppression in stage 5, all cycles	4	3–5
Rate of viral rebound cycle 1, all stages	0.4	0.3–0.5
Rate of viral rebound cycle 2 and 3, all stages	1.4	1.1–1.7
Fraction with viral rebound cycle 1, stage 1 to 3	0.6	0.5–0.7
Fraction with viral rebound cycle 1, stage 4 and 5	0.3	0.2–0.4
Fraction with viral rebound cycle 2 and 3, stage 1 to 5	0.5	0.4–0.6

### Fitting to Swiss HIV and AIDS Diagnosis Case Reports

We fitted the transmission model simultaneously to different sources of surveillance data from the Swiss Federal Office of Public Health. These included the numbers of new HIV diagnoses from 1995 to 2010, new AIDS cases from 1983 to 1996 and concurrent (within 60 days) HIV and AIDS diagnoses from 1983 to 2010. AIDS diagnoses after 1996 were not used to fit the model because these were affected by the use of cART. However, these data points could be used to verify the model predictions. Whilst mandatory reporting of positive HIV diagnoses in Switzerland dates back to 1988, we only used data from 1995 onwards because there was over-reporting of case numbers from laboratory reports in earlier years, which included multiple positive reports from the same individuals.

The numbers of infections acquired in Switzerland and abroad were estimated from information collected at HIV diagnosis in surveillance case reports (2007 onwards) and from the Swiss AIDS Transmission (CHAT) study in 2005 and 2006 [25], [26]. Of 814 MSM included in these data sources, 18% were reported to have acquired their infection abroad, and 62% from a partner in Switzerland (20% of responses missing). Of those reporting the country of infection, 84% of men with Swiss nationality and 66% of those with foreign nationality were reported to have been infected in Switzerland. Overall, we estimated that 77% of HIV infections amongst MSM were acquired in Switzerland.

The model was solved numerically using a Runge-Kutta 4 algorithm and fitted using maximum likelihood methods [19], [20]. To define the likelihood, we assumed that numbers of HIV and AIDS cases were Poisson distributed around a mean defined by the model as in Bezemer et al. [20]. For convenience, instead of maximising the likelihood, we minimised the equivalent deviance measure. We used the downhill simplex optimisation algorithm to find the minimum. The algorithm was started from various starting values to ensure that the optimisation was robust and that local optima were avoided. The fitting procedure resulted in the estimation of twelve unknown parameters (with 95% confidence intervals, CI) that are used to interpret the epidemic. The analysis of the model was done in different time periods, as defined by Bezemer and colleagues [19], [20]: 1980–1983; 1984–1994; 1995–1999; 2000–2004. In this study, the current cART era was considered as the most recent period, 2005–2010. The epidemic was assumed to have started with cases imported from abroad in 1980. The number of imported cases was estimated in three different time periods: 1980–1983, 1984–2004, and 2005–2010.

### Model Outputs

We used the best-fit model to estimate the following outputs: number of new HIV infections in each calendar year; proportion of infected patients remaining undiagnosed; proportion of new infections transmitted by undiagnosed patients; HIV diagnosis rate; net transmission rate; reproduction number.

The HIV diagnosis rate was set at zero from 1980–1983 because there was no serological test. HIV diagnosis was assumed to occur within one month of developing AIDS. It was also assumed that no HIV diagnosis is possible during primary infection when the antibody response has not fully developed yet. The mean time to diagnosis was calculated from the diagnosis rate. The net transmission rate, β(t), measures the rate at which an infectious HIV-positive individual infects new individuals, relative to the situation at the beginning of the epidemic [19], [20]. This parameter is an approximate measure of the risk behaviour rate as it incorporates changes in the partner change rate, in sexual risk-taking behaviours such as lack of condom use or serosorting, and the effects on transmissibility of co-existing sexually transmitted infections. The transmission rate was assumed to be the same for infections acquired in Switzerland and abroad. Other factors affecting transmissibility related to awareness of diagnosis, stage of infection and treatment were already taken into account in the model. An important assumption is that the overall incidence is not affected by epidemic saturation effects, which means that the transmission rate can be averaged across heterogeneous groups with different risk behaviours. The reproduction number, R(t), is the average number of people a man infected with HIV in Switzerland at time t would infect over his whole infectious lifespan if conditions remained the same at time t [27]. R(t) has to be below 1 for the epidemic to be controlled. Note that in the presence of infections acquired abroad, incidence can increase even when R(t)<1, especially when R(t) is close to 1. These increases are much slower than when R(t)>1, when the epidemic grows exponentially. R(t) is calculated from the best fit values of parameters describing infectiousness, diagnosis rates and the net transmission rate.

### Hypothetical Scenarios

We used the model to explore the effects of changing certain input parameters to represent different kinds of interventions. We implemented an intervention based on ‘Mission Possible’ with 10%, 50%, or 100% of MSM having 3 months of safe sex (assuming the net transmission rate to be zero) followed by an HIV test, every year starting in 2011. We also implemented a scenario with annual HIV testing, on average, and immediate treatment, i.e. ‘test and treat’ [28] (assuming an exponential time to the next test with mean one year). We compared these two prevention scenarios with two other scenarios in which the net transmission rate was reduced to the levels of the 1984–1994 or the 1995–1999 historical periods.

### Multivariate Sensitivity Analysis

We added to the basic transmission model of Bezemer et al. [19], [20] a multivariate sensitivity analysis to investigate the impact of assumptions about input parameters on the model predictions using a method that extends the Latin Hypercube sampling method [29]. For each of the 45 fixed input parameters, we identified a range of plausible values based on literature or on data from the SHCS cohort (Figure 1, Table 1, Table S1). We partitioned each parameter into 250 equidistant possible values spanning its whole plausible range. The sensitivity of the model to its fixed input parameters was then evaluated by sampling from the range of possible parameter values. Parameter values were sampled from a Latin Hypercube such that each possible value was sampled exactly once. We extended the Latin hypercube sampling method [29] by refitting the model to the data for each parameter set and re-estimating the twelve unknown parameters; in this way we explore a wide range of input parameters, but only with the restricted set of scenarios that best fit the epidemic data.

Partial rank correlation coefficients (PRCCs) were calculated for the correlation between each input parameter and the net transmission rate β(t). In general, PRCC values near 1 (or −1) indicate a strong positive (or negative) influence of the input parameter on β(t), whilst values near 0 indicate little influence. We conducted the sensitivity analysis for the main results and the hypothetical scenarios.

## Results

**Figure 2:**
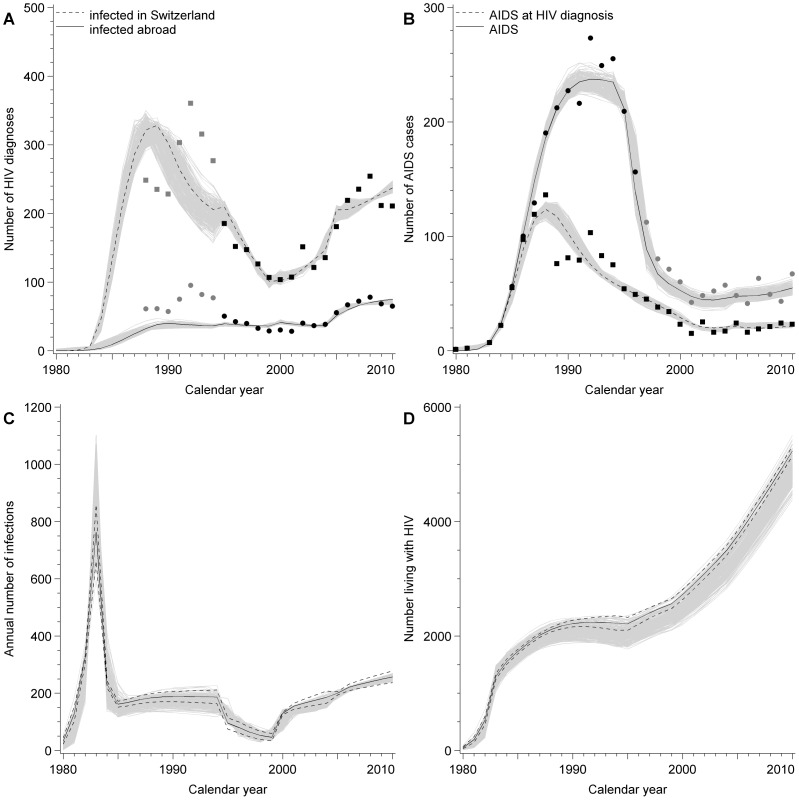
Model fits and estimates. Model fits to reported HIV and AIDS cases. Panel A) annual HIV diagnoses separated into infections acquired in Switzerland (black filled squares, dashed line) and abroad (black filled circles, solid line); B) annual number of new AIDS cases (black filled circles, solid line) and concurrent HIV and AIDS diagnoses (squares, black dashed line). Model estimates: C) annual number of new infections in Switzerland, D) number of men who have sex in Switzerland living with HIV. Continuous lines show model fit; dashed lines in panels C and D are 95% confidence intervals. Solid shapes show reported data; black shapes are data points used for fitting, grey shapes are not used for fitting. Grey lines show results of multivariate sensitivity analyses.

**Figure 3:**
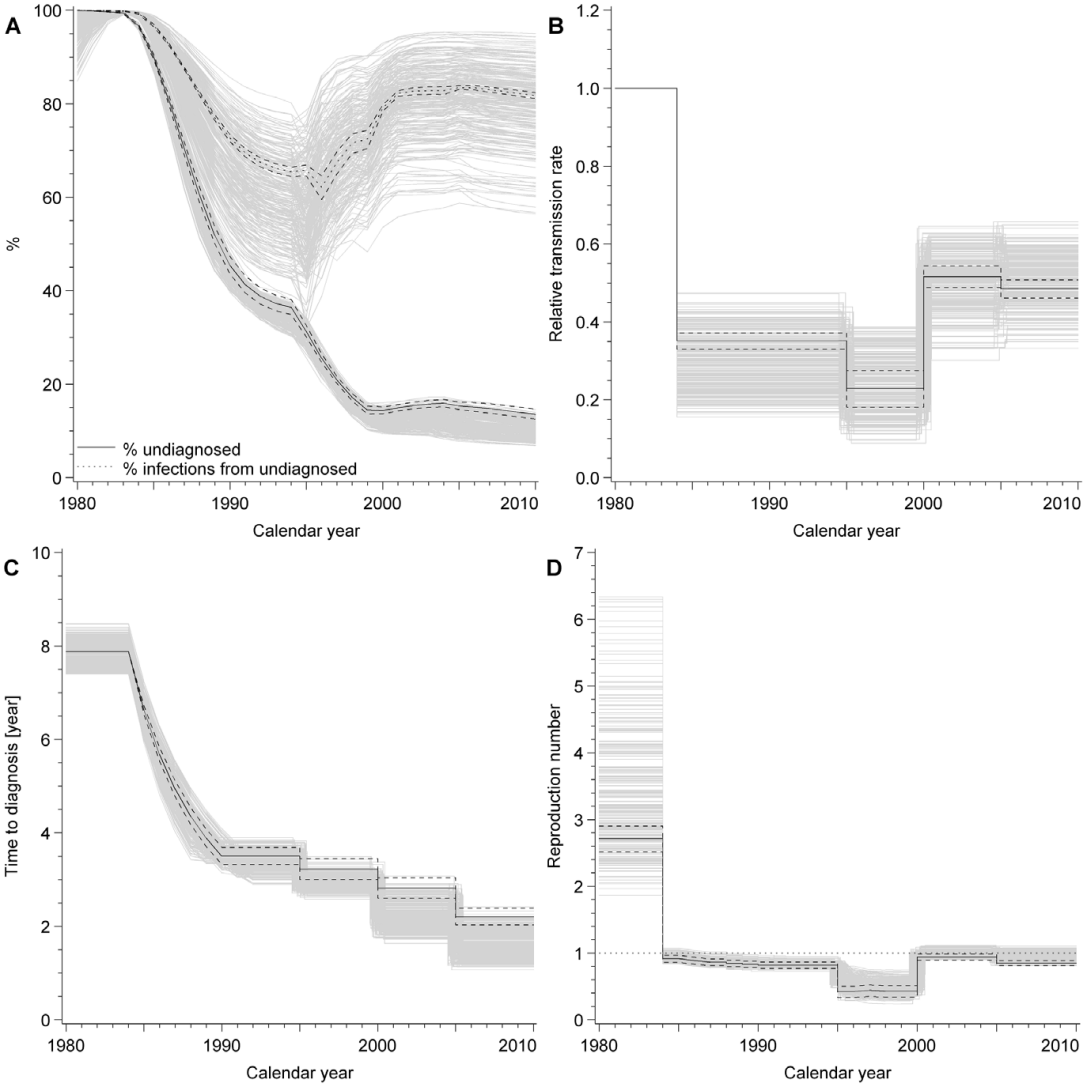
Model estimates for unknown parameters. A) percentage of infected patients remaining undiagnosed (solid line, dashed lines show 95% confidence interval) and percentage of new infections transmitted by those remaining undiagnosed (dotted line, dashed lines show 95% confidence interval); B) net transmission rate relative to the period 1980–1983 (dotted line, dashed lines show 95% confidence interval); C) average time from HIV infection to diagnosis in years (dotted line, dashed lines show 95% confidence interval); D) reproduction number (dotted line, dashed lines show 95% confidence interval). Grey lines show results of multivariate sensitivity analyses.

Using the best-fit model, we produced curves of the numbers of diagnosed HIV infections and AIDS cases in MSM that fitted the observed data well, including reported AIDS cases from 1996 onwards, which were not used for fitting (Figures 2A and 2B). Figure 2C shows the predicted numbers of incident HIV infections in MSM in Switzerland. According to the best-fit model, this peaked in 1983 with approximately 800 new infections, reached a nadir of about 50 in 1999 and has continued to increase since then. Figure 2D shows that the estimated number of MSM living with HIV remained at about 2000 for almost a decade (1987 to 1998). The total number of MSM estimated to be living with HIV in Switzerland has steadily increased during the cART era and more than doubled to 5229 (95% CI 5147, 5313). We estimated that by the end of 2010, 13.5% (95% CI 12.5, 14.6%) of all HIV-infected MSM had not yet been diagnosed and were thus unaware of their infection (Figure 3A). These MSM accounted for 81.8% (95% CI 81.1, 82.4%) of new HIV infections (Figure 3A).

The changes in the net transmission rate and time to diagnosis that we estimated using the model are shown in Figure 3B and 3C. Figure 3D shows corresponding changes in the estimated reproduction number. The highest transmission rate occurred in the earliest phase from 1980–1983 when there was no HIV diagnosis or treatment; during this phase the reproduction number R(t) was estimated to be 2.72 (95% CI 2.52, 2.90). The largest decrease in the net transmission rate occurred from 1984–1994, associated with the introduction of HIV testing and the assumption of reduced sexual risk taking after receiving an HIV diagnosis. During this phase the transmission rate fell by an estimated 65% (95% CI 63, 67%), the mean time from infection to diagnosis decreased to 3.5 (95% CI 3.3, 3.7) years and the reproduction number declined to 0.82 (95% CI 0.77, 0.86), which is below the epidemic threshold. This resulted in a plateau in the observed annual number of new infections at around 200 per year from 1984–1994 (Figure 2C).

In the early cART period from 1995–2000, the net transmission rate and the reproduction number (0.43, 95% CI 0.34, 0.51) were at their lowest (Figure 3B, 3D). From 2000–2005 the transmission rate increased to 49% (95% CI 46, 51%) of its initial value in 1980–1983, resulting in an increase in the reproduction number to 0.85 (95% CI 0.81, 0.89). This increase in transmission rate was observed for each of the 250 parameter sets in the multivariate sensitivity analysis. From 2005–2010, there were small reductions in the net transmission rate and reproduction number. Although the reproduction number has been below the epidemic threshold since 2000, the estimated number of annual new infections has continued to increase, to more than 250 in 2010 (Figure 2C).

### Hypothetical Scenarios

**Figure 4:**
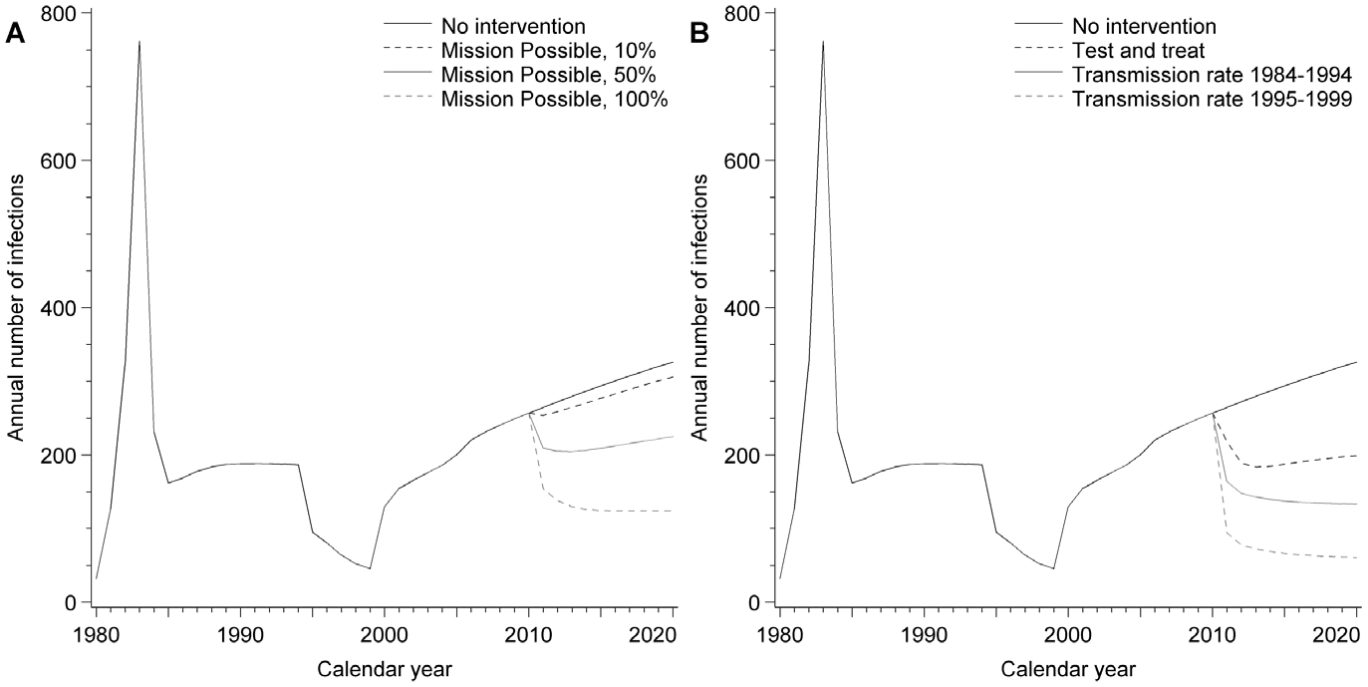
Hypothetical scenarios for the potential development of the HIV epidemic in Switzerland. Annual HIV incidence: A) without intervention and under various ‘Mission Possible’ scenarios with 10%, 50%, or 100% of MSM having safe sex for 3 months each year starting in 2011, followed by an HIV test; B) without intervention, under a ‘test and treat’ strategy (assuming that the average time between infection and diagnosis is reduced to 1 year and treatment is started immediately), or assuming that the net transmission rate falls to the levels attained in 1984–1994 or 1995–1999.

The potential development of the HIV epidemic in MSM in Switzerland from 2011 onwards under different scenarios is shown in Figures 4A and 4B. If conditions do not change the incidence would carry on rising to more than 300 infections per year, which would result in 8654 MSM living with HIV by 2020. ‘Mission Possible’ scenarios repeated yearly with 10%, 50%, and 100% of MSM having safe sex for 3 months followed by an HIV test, would reduce HIV incidence below the 2010 level and prevent 167, 882, and 1673 new infections, respectively by 2020 (Figure 4A). A strategy of ‘test and treat’ in which the average time from infection to diagnosis is reduced to 1 year, assuming cART is started immediately and viral suppression is achieved within one month after diagnosis, would reduce HIV incidence to 199 infections per year by 2020 thus preventing 1028 new infections. More marked reductions in HIV incidence would be achieved if the risk behaviour rate fell to the levels attained in 1984–1994 or 1995–1999 (Figure 4B); the annual HIV incidence would fall to 133 and 61 new infections, respectively in 2020 (1562 and 2273 new infections prevented by 2020), and the reproduction number to 0.62 and 0.40, respectively. However, under all considered scenarios the number of MSM living with HIV would continue to increase due to the decreased mortality since the advent of cART.

### Multivariate Sensitivity Analysis

The parameters related to disease progression and the relative infectiousness of the different stages of the infection had the largest influence on the estimates of the net transmission rate β(t) (Table S2). In particular, PRCC ranged from −0.85 to −0.94 for the relative infectiousness of primary infection indicating that, for higher values of this parameter, the estimated net transmission rate decreased. Similarly, PRCC ranged between 0.88 and 0.94 for the reciprocal of the duration of primary infection, which is the rate *α_P_* at which infected individuals move from primary infection to stage 1 (Figure 1, Table S2). To understand these associations it should be noted that, for higher values of *α_P_*, the duration of primary infection gets shorter and this is compensated for by a higher estimate of the transmission rate in order to generate a certain number of HIV diagnoses and AIDS cases. Analogously, a higher infectiousness of primary infection is compensated for by lower estimates of β(t).

## Discussion

This study shows that the HIV epidemic amongst MSM in Switzerland has been increasing since the year 2000. The model suggests that the increase in new infections results predominantly from an increase in the transmission rate, and that the majority of new infections are transmitted by MSM unaware of their infection. Testing rates have increased steadily over the period of the epidemic and the time to diagnosis has decreased.

The main strength of this study is the use of empirical data from a national surveillance system and clinical cohort, which could be adapted for use using a previously developed model [19], [20]. The model, previously used in the Netherlands, could be used in Switzerland because both countries have HIV epidemics that are concentrated in MSM and both countries have HIV surveillance systems that collect data on new HIV diagnoses acquired within country and abroad, new AIDS diagnoses and simultaneous HIV and AIDS diagnoses. An additional strength of the current analysis is the multivariate sensitivity analysis, which supplements the earlier univariable sensitivity analysis for the model [20] and extends the widely used Latin hypercube sampling method [29].

There are limitations to our study related to biases in the surveillance data. Our reconstruction of the earlier years of the epidemic is not very precise because the numbers of HIV case reports before 1995, which were known to be unreliable, were not used for fitting the model. Nevertheless, the predicted numbers fitted the observed data for new HIV diagnoses from 1995 onwards and for AIDS diagnoses well. The model predictions might not capture trends in the epidemic if sex between men as the route of transmission has been substantially under-reported or if HIV-infected MSM who are not registered by the surveillance system (because they die or emigrate without being registered) are an important source for transmission. We do not have any information to support or refute these possible biases. The model predictions of HIV prevalence are affected by considering only HIV-related deaths and by ignoring loss to follow-up. The number of MSM living with HIV infection (Figure 2D) can be considered an upper bound on the true number.

The most important assumption when interpreting the outcomes of the model is that the transmission parameter β(t) can be considered as an average measure of risk behaviour. This assumption is valid in countries like Switzerland with low HIV prevalence where saturation effects do not affect the dynamics of transmission. Whilst there are no representative HIV seroprevalence studies in Switzerland, estimates from Europe suggest a prevalence of 6% or lower [30]. In addition, gene sequencing studies have identified clusters of HIV infections showing evidence of recent transmission amongst MSM, which suggest that saturation has not occurred [2]. Another assumption was that there is no transmission when viral load is below the level of detection, defined as <1000 copies/ml. This allowed data from the 1990s to be included when 1000 copies/ml was the cut-off of the viral load assays that were then available. We believe that the transmission rate at this level of viraemia will come from a very small group of patients; first because no transmission events were observed <400 copies/ml in a meta-analysis of treated patients [7] and second, most patients will actually have a viral load <50 copies/ml. A further limitation is that the model does not produce reliable estimates of the proportions of HIV transmission occurring during acute primary infection and asymptomatic infection. This would be a valuable output but it could not be formulated in the model because of a lack of reliable data about risk-changing strategies like partner change rates, serosorting, and serial monogamy at different stages of infection. As a result of such strategies, the infectiousness of primary infection relative to the asymptomatic undiagnosed phase may effectively be lower than as parameterised in our model.

This study is the first to reconstruct the HIV epidemic in Swiss MSM using a mathematical model. The model predictions strongly suggest a resurgence of HIV transmission amongst MSM in Switzerland despite increasing levels of HIV testing and use of cART. The predicted decrease in the average time from HIV infection to diagnosis is supported by results from the Swiss Gay Survey 2009, which shows that the estimated proportion of MSM who had had an HIV test within the last 12 months increased from 30% in 2007 to 40% in 2009 [6], and the SHCS in which the median CD4 cell count at diagnosis in MSM has increased from 387 cells/mm^3^ (inter-quartile range, 160–521) in 2000 to 441 cells/mm^3^ (286–598) in 2009. An average of 2.2 years between infection and diagnosis would still allow for a substantial amount of HIV transmission, especially if treatment does not start immediately and if the reduction in risky sexual behaviour following HIV diagnosis is less than in earlier years [23].

The model outputs suggest that the increase in HIV transmission amongst MSM in Switzerland is the result of continuing risky sexual behaviour, particularly by those who are unaware that they are HIV-infected. In both the Dutch and Swiss studies the model predicted a high proportion of new infections attributable to transmission from undiagnosed MSM (in 2006, 90% in the Netherlands [20], Swiss model 83%). However, despite an increase in risky sexual behaviour, the within-country reproduction number is predicted to be below the epidemic threshold from 2005–2010. Still, in the Swiss model, numbers of new HIV infections are predicted to increase, which is likely to be due to infections acquired abroad. About 20% of new HIV infections in MSM in Switzerland are acquired abroad (Figure 2), compared with about 7% in the Netherlands [31], where local transmission appeared sufficient to sustain transmission [20]. Within Switzerland, there is some empirical support for a recent increase in sexual risk behaviour amongst MSM. The odds of having unprotected sex among HIV-infected MSM from the SHCS with both stable and occasional partnerships increased between 2007 and 2009 [14], whereas an earlier study found no evidence of an increase in unsafe sexual behaviour from 2000 to 2003 amongst all transmission groups combined [32]. Analyses from the Swiss Gay Survey have found reductions in some safer sex behaviours from 1992 to 2004 [13] and, in the 2007 survey, high prevalences of withdrawal before ejaculation, serosorting, and strategic positioning amongst MSM reporting unprotected anal sex with casual partners [33]. Increases in sexual risk behaviour have further been associated with HIV optimism following the introduction of cART [4], [34], [35].

The predictions from this study have implications for future prevention strategies. The greatest fall in the HIV transmission rate occurred during a time when testing and treatment were introduced, but when there were still frequent visible reminders of AIDS and its consequences, which probably reinforced the need for reduced sexual risk behaviour [36]. The gains predicted in the hypothetical ‘test and treat’ scenario that halves the average time to diagnosis from the 2010 level rely on the assumption that risk behaviour levels are constant or decline. The model estimates of an increasing transmission rate in the cART era, however, support the prediction that the prevention benefits of cART could be offset by increases in risk behaviour in untreated individuals [18]. Modest levels of uptake (10%) of a campaign like Mission Possible could result in a small transient reduction in new HIV diagnoses if repeated every year. Accurate diagnosis of primary HIV infection would be an important advance and interventions to reduce the proportion of undiagnosed individuals and attain long-term reduction of sexual risk behaviours should be pursued. Long term reductions in the number of new HIV infections in MSM in Switzerland will require increased and sustained uptake of effective interventions.

## Supporting Information

Table S1 **Parameters used in the mathematical model for HIV transmission amongst men who have sex with men (MSM) in Switzerland.** Durations are given in years, rates are per year. Years are continuous variables with whole years starting on 1 January.
(DOCX)

Table S2 **Partial rank correlation coefficients for the correlation between each input parameter and the net transmission rate β(t) amongst men who have sex with men in Switzerland.**
(DOCX)
