# Supplementary material for: Resurgence of HIV Infection among Men Who Have Sex with Men in Switzerland: Mathematical Modelling Study
Source: PLoS One. 2012 Sep 14;7(9):e44819. doi: 10.1371/journal.pone.0044819 (PMC3443082; doi:10.1371/journal.pone.0044819)
Supplement: Table S1 — Parameters used in the mathematical model for HIV transmission amongst men who have sex with men (MSM) in Switzerland. Durations are given in years, rates are per year. Years are continuous variables with whole years starting on 1 January. (DOCX) [file pone.0044819.s001.docx]

**Table S1:** Parameters used in the mathematical model for HIV transmission amongst men who have sex with men (MSM) in Switzerland. Durations are given in years, rates are per year. Years are continuous variables with whole years starting on 1 January.

| **Parameter** | | **Value** | **Range** | | **Description** |  |
| --- | --- | --- | --- | --- | --- | --- |
| *n_s_* | | 5 | - | | Number of disease stages [19] |  |
| 1*/α* | | 1.89 | 1.8 – 2.0 | | Duration of each disease stage [19] |  |
| 1*/α_P_* | | 0.24 | 0.10 – 0.5 | | Duration of primary infection [21] |  |
| *ρ_P_* | | 2.76 | 1.31 – 5.09 | | Relative infectiousness primary infection [21] |  |
| *ρ_s_* (*s*=1,..,4) | | 0.106 | 0.076 – 0.133 | | Relative infectiousness asymptomatic infection stage 1 to 4 [21] |  |
| *ρ_5_* | | 0.36 | 0.20 – 0.61 | | Relative infectiousness stage 5 (AIDS) [21] |  |
| *σ_0_* | | 0.47 | 0.37 – 0.57 | | Reduction factor of relative infectiousness after diagnosis pre- 1996 [22,23] |  |
| *σ_1_* | | 0.85 | 0.37 – 1.0 | | Reduction factor of relative infectiousness after diagnosis post-1996 [23] |  |
| *δ_s_(t)* (*s*=1,…,4) | |  |  | | Diagnosis rate stage 1 to 4, estimated in 4 time intervals (0 if *t* < 1984)^a^ |  |
| *δ_5_* | 12^*^ | | | 6 - 24 | Diagnosis rate at stage 5 (AIDS) | |
| *f_P_* | 1^*^ | | | 0 – 1 | Proportion of imported cases in primary infection | |
| *T_bs_* (s=1,…,3) | 1996^†^ | | | [-1.0, 1.0] | Year start treatment build-up stage 1 to 3 | |
| *T_b4_* | 1995^†^ | | | [0, 1.0] | Year start treatment build-up stage 4 | |
| *T_b5_* | 1995^†^ | | | [0, 0.5] | Year start treatment build-up stage 5 | |
| *T_fs_* (s=1,…,3) | 1998^†^ | | | [-1.0, 1.0] | Year stop treatment build-up stage 1 to 3 | |
| *T_f4_* | 1998^†^ | | | [-1.0, 1.0] | Year stop treatment build-up stage 4 | |
| *T_f5_* | 1996^†^ | | | [-0.5, 1.5] | Year stop treatment build-up stage 5 | |
| *γ_s_(t)* (*s*=1,...,3) | 0.7^†^ | | | 0.6 – 0.8 | Rate of achieving viral suppression cycle 1, stage 1, 2, and 3 (0 if *t* ≤ *T_bs_*, *γ_s_(t)* if *t* > *T_fs_*)^b^ | |
| *γ_4_(t)* | 2.8^†^ | | | 2.5 – 3.2 | Rate of achieving viral suppression cycle 1, stage 4 (0 if *t* ≤ *T_b4_*, *γ_4_(t)* if *t* > *T_f4_*)^b^ | |
| *γ_5_(t)* | 4^†^ | | | 3 – 5 | Rate of achieving viral suppression cycle 1, stage 5 (0 if *t* ≤ *T_b5_*, *γ_5_(t)* if *t* > *T_f5_*)^b^ | |
| *ι_2s_*_,_ *ι_3s_* (s=1,…,3) | 0.7^†^ | | | 0.6 – 0.8 | Rate of achieving viral suppression cycle 2 and 3, stage 1 to 3 | |
| *ι_24_*_,_ *ι_34_* | 2.8^†^ | | | 2.5 – 3.2 | Rate of achieving viral suppression cycle 2 and 3, stage 4 | |
| *ι_25_*_,_ *ι_35_* | 4^†^ | | | 3 – 5 | Rate of achieving viral suppression cycle 2 and 3, stage 5 | |
| *τ_1s_* (*s*=1,…,3) | 0.6^†^ | | | 0.5 – 0.7 | Fraction with viral rebound cycle 1, stage 1 to 3 | |
| *τ_14_*, *τ_15_* | 0.3^†^ | | | 0.2 – 0.4 | Fraction with viral rebound cycle 1, stage 4 and 5 | |
| *τ_2s_*, *τ_3s_* (*s*=1,…,5) | 0.5^†^ | | | 0.4 – 0.6 | Fraction with viral rebound cycle 2 and 3, stage 1 to 5 | |
| *κ_1s_* (*s*=1,…,5) | 0.4^†^ | | | 0.3 – 0.5 | Rate of viral rebound cycle 1, stage 1 to 5 | |
| *κ_2s_*, *κ_3s_* (*s*=1,…,5) | 1.4^†^ | | | 1.1 – 1.7 | Rate of viral rebound cycle 2 and 3, stage 1 to 5 | |
| *t_1_* | 1983 | | | - | Time interval 1 (1980 – *t_1_*) | |
| *t_2_* | 1989 | | | [-2.0, 2.0] | Time interval 2 (*t_1_*+1 – *t_2_*) | |
| *t_3_* | 1994 | | | [-0.5, 0.5] | Time interval 3 (*t_2_*+1 – *t_3_*) | |
| *t_4_* | 1999 | | | [-0.5, 0.5] | Time interval 4 (*t_3_*+1 – *t_4_*) | |
| *t_5_* | 2004 | | | [-0.5, 0.5] | Time interval 5 (*t_4_*+1 – *t_5_*) | |
| *t_6_* | 2010 | | | - | Time interval 6 (*t_5_*+1 – 2010) | |
| *β(t)* |  | | |  | Net per infectious capita transmission rate, estimated in 5 time intervals | |
| *A_0_* |  | | |  | Number of imported cases 1980–1983 | |
| *A_1_* |  | | |  | Number of imported cases 1984–2004 | |
| *A_2_* |  | | |  | Number of imported cases 2005–2010 | |

^*^ assumption;

^†^ based on data from the Swiss HIV Cohort Study;

^a^ linear interpolation between 1984 and 1990;

^b^ rates of achieving viral suppression in cycle 1 are time-dependent and increase linearly between 0 and their maximum value.
