# Supplementary material for: Resurgence of HIV Infection among Men Who Have Sex with Men in Switzerland: Mathematical Modelling Study
Source: PLoS One. 2012 Sep 14;7(9):e44819. doi: 10.1371/journal.pone.0044819 (PMC3443082; doi:10.1371/journal.pone.0044819)
Supplement: Table S2 — Partial rank correlation coefficients for the correlation between each input parameter and the net transmission rate β(t) amongst men who have sex with men in Switzerland. (DOCX) [file pone.0044819.s002.docx]

**Table S2:** Partial rank correlation coefficients for the correlation between each input parameter and the net transmission rate *β(t)* amongst men who have sex with men in Switzerland.

| **Parameter** | **Correlation with net transmission rate *β(t)*, by time period** | | | | |
| --- | --- | --- | --- | --- | --- |
|  | **1980–1983** | **1984–1995** | **1996–1999** | **2000–2004** | **2005–2010** |
| *α_P_* | ^**^0.91 | ^**^0.94 | ^**^0.88 | ^**^0.95 | ^**^0.94 |
| *α* | 0.04 | ^**^0.43 | 0.07 | -0.05 | -0.02 |
| *σ_0_* | -0.05 | ^**^-0.52 | ^**^-0.50 | 0.01 | -0.03 |
| *σ_1_* | 0.10 | ^**^0.23 | ^**^-0.60 | ^**^-0.51 | ^**^-0.43 |
| *ρ_P_* | ^**^-0.93 | ^**^-0.89 | ^**^-0.85 | ^**^-0.94 | ^**^-0.93 |
| *ρ_s_* (*s*=1,..,4) | ^**^-0.19 | ^**^-0.75 | ^**^-0.67 | ^**^-0.59 | ^**^-0.50 |
| *ρ_5_* | 0.06 | ^**^-0.67 | ^**^-0.78 | ^**^-0.28 | ^**^-0.19 |
| *f_P_* | ^**^-0.37 | ^**^-0.20 | ^**^-0.68 | ^**^-0.50 | ^**^-0.67 |
| *γ_s_(t)* (*s*=1,...,3) | 0.08 | -0.12 | -0.11 | -0.05 | 0.00 |
| *γ_4_(t)* | 0.05 | -0.05 | ^**^-0.21 | -0.02 | 0.00 |
| *γ_5_(t)* | -0.07 | 0.03 | 0.15 | -0.01 | -0.01 |
| *t_2_* | -0.06 | ^**^0.21 | -0.14 | -0.13 | -0.09 |
| *t_3_* | 0.02 | -0.10 | -0.13 | 0.03 | 0.00 |
| *t_4_* | 0.09 | 0.10 | ^**^0.64 | 0.18 | 0.04 |
| *t_5_* | -0.02 | 0.01 | 0.01 | 0.07 | -0.07 |
| *τ_1s_* (*s*=1,…,3) | -0.02 | -0.04 | 0.04 | -0.14 | -0.11 |
| *τ_14_* | -0.01 | 0.00 | 0.07 | -0.07 | -0.04 |
| *τ_15_* | -0.08 | 0.05 | -0.05 | -0.15 | -0.10 |
| *τ_2s_* (*s*=1,…,3) | -0.03 | 0.07 | ^**^0.26 | 0.01 | -0.01 |
| *τ_24_* | 0.02 | -0.06 | 0.04 | -0.04 | -0.02 |
| *τ_25_* | -0.09 | -0.09 | -0.09 | -0.13 | -0.11 |
| *τ_3s_* (*s*=1,…,3) | -0.10 | -0.05 | 0.02 | -0.05 | -0.09 |
| *τ_34_* | -0.06 | -0.05 | 0.05 | -0.13 | -0.07 |
| *τ_35_* | -0.02 | 0.06 | 0.05 | 0.02 | 0.01 |
| *κ_1s_* (*s*=1,…,3) | 0.02 | 0.00 | -0.09 | -0.06 | -0.06 |
| *κ_14_* | -0.03 | 0.02 | 0.01 | 0.01 | 0.02 |
| *κ_15_* | 0.09 | -0.02 | ^**^-0.21 | -0.07 | -0.02 |
| *κ_2s_* (*s*=1,…,3) | -0.07 | 0.09 | 0.08 | 0.01 | 0.01 |
| *κ_24_* | -0.04 | -0.02 | -0.01 | 0.02 | 0.01 |
| *κ_25_* | -0.04 | -0.12 | -0.15 | -0.08 | -0.07 |
| *κ_3s_* (*s*=1,…,3) | 0.05 | -0.01 | 0.02 | 0.11 | 0.12 |
| *κ_34_* | 0.06 | 0.08 | 0.05 | 0.05 | 0.07 |
| *κ_35_* | -0.07 | -0.03 | -0.05 | -0.10 | -0.10 |
| *ι_2s_* (s=1,…,3) | 0.00 | 0.05 | 0.06 | 0.05 | 0.04 |
| *ι_24_* | -0.03 | 0.08 | 0.10 | -0.05 | -0.06 |
| *ι_25_* | -0.05 | 0.04 | 0.12 | 0.02 | 0.01 |
| *ι_3s_* (s=1,…,3) | 0.11 | 0.06 | 0.05 | 0.04 | 0.03 |
| *ι_34_* | -0.01 | -0.08 | -0.06 | 0.00 | -0.01 |
| *ι_35_* | 0.15 | 0.05 | 0.12 | 0.16 | 0.15 |
| *T_bs_* (s=1,…,3) | 0.05 | 0.01 | 0.24 | 0.04 | 0.05 |
| *T_b4_* | -0.06 | ^**^-0.33 | ^**^0.45 | -0.02 | -0.04 |
| *T_b5_* | 0.12 | 0.12 | -0.06 | 0.16 | 0.14 |
| *T_fs_* (s=1,…,3) | -0.10 | -0.07 | -0.03 | -0.02 | -0.03 |
| *T_f4_* | -0.03 | -0.14 | 0.13 | -0.11 | -0.10 |
| *T_f5_* | 0.07 | 0.04 | ^**^-0.25 | 0.05 | 0.03 |

^**^*p*<0.001; ^*^*p*<0.01
